# Effect of Hop-Stabilization Training on Ankle Instability and Function of Adolescent Female Basketball Players with Chronic Ankle Instability: A Double-Blind, Prospective, Cluster-Randomized Controlled Trial

**DOI:** 10.3390/jcm14103502

**Published:** 2025-05-16

**Authors:** Han-Soo Park, Jae-Keun Oh, Ye-In Hong, Jun-Young Kim, Jin-Ho Yoon

**Affiliations:** Sports Medicine Laboratory, Korean National Sport University, Seoul 05541, Republic of Korea; 302673@knsu.ac.kr (H.-S.P.); sportsomd@knsu.ac.kr (J.-K.O.); 20yen12@naver.com (Y.-I.H.)

**Keywords:** adolescent, balance training, basketball, functional ankle instability, hop-stabilization training, neuromuscular control

## Abstract

**Background/Objectives:** Adolescent female basketball players are frequently affected by lateral ankle sprains that may progress to chronic ankle instability (CAI) if not adequately managed. This double-blind, prospective, cluster-randomized controlled trial aimed to compare the effects of hop-stabilization training (hop training) and those of traditional balance training on ankle instability and functional performance of this population. **Methods:** Thirty-two adolescent female basketball players with CAI were cluster-randomized into the hop training group (HG; *n* = 16) or balance training group (BG; *n* = 16). Participants completed three 20 min sessions per week for 6 weeks. The hop training protocol comprised multiplanar hopping exercises with progressive increases in the landing volume and an emphasis on controlled landing mechanics. The balance training protocol included single-leg stance and basketball-specific dynamic activities with gradually increasing difficulty. Primary outcomes were self-reported ankle stability (Cumberland ankle instability tool [CAIT] score) and performance test results (*t*-test, lateral hop test, figure-8 hop test results). Secondary outcomes included static and dynamic balance and isometric ankle strength (dorsiflexion [DF], plantar flexion, inversion, eversion [EV]). Assessments were conducted at baseline and after interventions. **Results:** The HG and BG exhibited significant improvements in CAIT scores and balance. However, the HG demonstrated significantly greater enhancements in dynamic performance test results and notable improvements in DF and EV strength compared to those of the BG. **Conclusions:** Hop training comprising the close replication of the multidirectional and dynamic demands of basketball was more effective than traditional balance training for enhancing functional performance and ankle strength.

## 1. Introduction

Adolescent female basketball players frequently experience lateral ankle sprains caused by repetitive jumping, rapid directional changes, and frequent pivoting maneuvers [1]. These high-impact movements place considerable stress on the ankle joint, thus increasing the likelihood of sprains and subsequent residual symptoms [2,3]. Acute ankle sprains that are not adequately managed may progress to chronic ankle instability (CAI) [4], which encompasses both structural alterations and sensorimotor deficits. CAI is often manifested as recurrent sprains or the sensation of “giving way” during dynamic activities, thus reflecting underlying deficits in proprioception, neuromuscular control, and, in some cases, ligamentous integrity [5]. These sensorimotor deficits are characterized by impaired joint position sense, reduced reflex excitability, and diminished dynamic stabilization during sport-specific tasks [6,7,8]. Consequently, when severe mechanical instability is not present, rehabilitation protocols that emphasize proprioception and functional stability tend to be more effective for managing CAI [9].

Neuromuscular and balance-oriented approaches can address these deficits effectively, thereby reducing the risk of recurrent ankle sprains [10]. In particular, balance training has demonstrated significant benefits for improving postural control and lowering the repeat injury rates of athletes with CAI [11]. Well-designed balance protocols enhance proprioceptive feedback, refine neuromuscular coordination, and reinforce sensorimotor integration, leading to stronger dynamic joint stability [12,13]. However, recent evidence suggested that hop-stabilization training (hop training), which emphasizes controlled landing mechanics and dynamic lower limb alignment, may offer additional benefits by more closely replicating the sport-specific demands of basketball [14]. By incorporating multiplanar hopping sequences and deliberate jump-landing drills, hop training fosters the neuromuscular adaptability essential for managing rapid changes in direction, force, and velocity [14]. These exercises not only enhance lower limb alignment during high-impact activities but also promote greater responsiveness and control throughout the kinetic chain [15]. Although direct evidence linking hop training to reduced injury rates is limited, its focus on controlled landing mechanics and dynamic stability suggests the potential for enhanced functional performance. Thus, hop training could be a valuable addition to comprehensive rehabilitation and performance enhancement programs for adolescent basketball players with CAI.

Despite the recognized benefits of both hop-stabilization and balance training, direct comparisons of these interventions for adolescent female basketball players with CAI exists are lacking. Because of the frequent jumping and rapid directional changes inherent in basketball, it has been hypothesized that hop training may yield greater improvements in ankle stability and functional performance by more closely mimicking sport-specific demands. Therefore, this double-blind, prospective, cluster-randomized controlled trial aimed to compare the effects of hop training and traditional balance training on ankle instability, performance, and functional outcomes of adolescent female basketball players with CAI.

We hypothesized that hop training would lead to greater improvements in ankle joint stability, sport-specific performance (assessed using the T-test, lateral hop test, and figure-8 hop test), and functional outcomes (including static and dynamic balance as well as ankle strength) compared to those achieved with traditional balance training. This hypothesis was based on the premise that because hop training more closely mimics the neuromuscular demands of basketball, it may provide superior sport-specific adaptation. The findings of this study could offer valuable guidance that may help optimize rehabilitation and performance enhancement strategies for this population. Specifically, from a biomechanical perspective, hop training likely enhances adaptive neuromuscular control through improved anticipatory muscle activation and optimized proprioceptive feedback during dynamic landing and directional movements that are commonly seen in basketball. Recent studies have shown that hop-stabilization exercises effectively improve dynamic postural stability, neuromuscular coordination, and proprioceptive responses, which are crucial for managing rapid directional changes and maintaining functional ankle stability during complex athletic movements [15,16]. These neuromuscular adaptations may contribute indirectly to reduced injury risks by enhancing athletes’ ability to maintain optimal joint alignment and dynamic balance during sport-specific tasks.

## 2. Materials and Methods

This double-blind, prospective, cluster-randomized controlled trial evaluated the effects of hop training on adolescent female basketball players with CAI. To minimize potential cross-contamination between participants and reduce selection bias, each basketball team was defined as a single cluster.

An independent statistician, who was not involved in participant recruitment, data collection, or analysis, performed the randomization procedure using the Research Randomizer software program (https://www.randomizer.org/, accessed on 5 December 2024). To minimize selection bias and potential cross-contamination, randomization was conducted at the cluster level, with each basketball team defined as a single cluster. Teams were randomly allocated in a 1:1 ratio to either the hop-stabilization training group (HG) or the balance training group (BG).

The intervention programs were administered by two designated investigators (J.Y.K. and Y.I.H.), who were solely responsible for delivering the training protocols. To ensure transparency and to avoid bias in outcome assessments, these investigators were not involved in data collection, outcome evaluation, or statistical analysis. The remaining research team members, who conducted these tasks, were blinded to group assignments throughout the study. This blinding protocol preserved the integrity of the double-blind design and minimized potential observer and performance biases.

**Figure 1:**
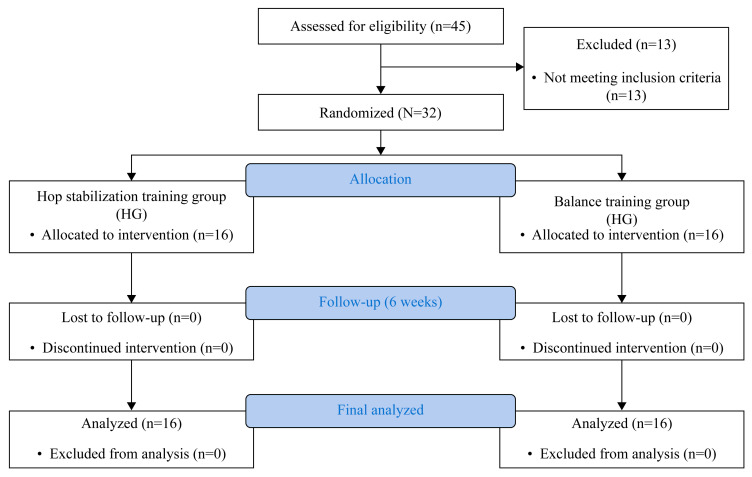
CONSORT flow diagram of participant recruitment, allocation, follow-up, and analysis.

This study adhered to the ethical standards outlined in the Declaration of Helsinki. Ethical approval was obtained from the Institutional Review Board of Korea National Sport University (approval no. 1263-202403-HR-042-02). Additionally, the trial was registered with the Clinical Research Information Service (registration no. KCT0009974, date of approval 29 November 2024). The Consolidated Standards of Reporting Trials (CONSORT) diagram is presented in Figure 1.

### 2.1. Participants

Adolescent female basketball players 13 to 18 years of age with CAI were recruited for this study. Eligibility criteria included a history of at least two previous lateral ankle sprains and active participation in basketball training and competition without current injury-related restrictions. Participants reported a self-perceived sensation of ankle instability or “giving way” in the affected ankle and had experienced an ankle sprain within the past 6 months that led to the interruption of training for more than 1 day.

The Cumberland ankle instability tool (CAIT) was used to confirm eligibility based on the CAI criteria. Participants with a CAIT score ≤ 25, which indicated clinically significant ankle instability, were included to ensure substantial functional impairment [17]. For participants with bilateral CAI, the ankle with the lower CAIT score was designated as the dominant side for intervention purposes.

Individuals who experienced a lower extremity fracture or underwent surgical intervention within 3 months prior to the study were excluded. Participants who had received any form of rehabilitation or treatment for ankle sprains, whether formal or informal, within 6 months before enrollment were also excluded. Additionally, individuals who exhibited severe mechanical instability, which was defined as grade III ligamentous laxity determined using the anterior drawer and talar tilt tests, were excluded from participation to ensure that the study sample primarily represented cases of CAI characterized by sensorimotor deficits rather than pronounced structural damage.

The sample size was calculated using the G*Power software program (version 3.1.9.7; Heinrich Heine University, Düsseldorf, Germany) and by referencing an effect size (f) of 0.25 reported by Ardakani et al. [14]. To achieve statistical power of 0.80 with a significance level (α) of 0.05, 32 participants were required after accounting for an anticipated 10% dropout rate. Accordingly, 32 eligible adolescent female basketball players were randomly assigned to one of two intervention groups (HG or BG).

Before enrollment, participants and their legal guardians received a detailed explanation of the study’s purpose, procedures, and potential risks. Written informed consent was obtained from all participants and guardians to confirm voluntary participation.

### 2.2. Intervention

The intervention protocols for this study consisted of hop training and balance training, which are two distinct training programs. Participants completed three 20 min intervention sessions per week for 6 weeks. These sessions were conducted on alternating days (Monday, Wednesday, and Friday, or Tuesday, Thursday, and Saturday) to allow adequate recovery. The hop training sessions were supervised and administered by one investigator (J.Y.K.), and the balance training sessions were conducted by another investigator (Y.I.H.). Both investigators were experienced practitioners with more than 3 years of athlete training experience, thus ensuring consistent adherence to the intervention protocols. Each session began with a standardized warm-up consisting of 5 min of free running and 10 min of static stretching to reduce the risk of injury and prepare participants for high-impact exercises. During the warm-up, participants were instructed to gradually increase their movement intensity to activate the lower limbs and improve joint mobility before engaging in the main exercises.

### 2.3. Hop Training

The hop training program included a variety of hopping exercises to replicate the multidirectional movements required during basketball. These exercises comprised side-to-side hops, forward and backward hops, figure-8 hops, zigzag hops, and four-square hops. The training volume was progressively increased to enhance participants’ endurance and adaptability to repetitive landing mechanics. Each session began with 160 landings during week 1. The number of landings increased by 40 each week and peaked at 320 landings during weeks 4 and 5; during the final week (week 6), this number was reduced slightly to 300 landings to prevent fatigue before postintervention assessments [14].

Proper landing mechanics were emphasized throughout the program to ensure that participants could stabilize their landings effectively. Key instructions included maintaining knee alignment over the toes, landing with flexed knees, and adopting a shoulder-width stance. Participants were encouraged to focus on stabilizing their landings before initiating the next hop to reduce the risk of improper alignment and injury.

During the final 3 weeks, exercises were performed at a speed of 2 Hz using a metronome to ensure a consistent rhythm and tempo. This rate required participants to complete two hops per second, thus promoting faster neuromuscular responses and simulating sport-specific conditions with rapid movements and directional changes. Rest periods were standardized at 30 s between sets and 1 min between exercises to maintain proper form and performance. To further enhance training effectiveness, real-time visual feedback was provided via mirrors, and oral feedback was provided by supervisors during each session while focusing on correcting knee valgus, landing posture, and foot positioning. The hop training program applied during this study is detailed in Table 1.

### 2.4. Balance Training

The balance training program implemented during this study was derived from a previously validated balance training protocol and systematically modified to align with the sport-specific demands of basketball players [11]. Each session included the following four primary exercises: single-leg stance; single-leg stance with leg swinging; single-leg squat (30–40°); and single-leg stance while performing basketball-related activities such as dribbling and passing. Two sets of these exercises were performed for 30 s per leg; a 30 s rest period between exercises was allowed to ensure training quality and minimize fatigue.

The progression of difficulty was structured over the 6-week intervention to optimize neuromuscular control and proprioceptive adaptation. During the initial phase, participants performed exercises on a stable surface with the eyes open to establish baseline postural stability. During the second week, the same exercises were conducted with the eyes closed to reduce reliance on visual feedback and enhance proprioceptive acuity. From the third week onward, participants transitioned to an unstable surface (e.g., a balance board) while maintaining visual input to further challenge sensorimotor control. During the final phase (weeks 4–6), single-leg stance exercises were performed on an unstable surface with the eyes closed to maximize proprioceptive engagement; however, dynamic movements such as basketball dribbling and passing were integrated with the eyes open to replicate sport-specific demands and facilitate functional neuromuscular adaptations.

Throughout the program, real-time feedback was provided by a supervising investigator to ensure correct posture, minimize knee valgus, and encourage proper weight distribution to avoid excessive compensatory movements. Gradual difficulty progression was emphasized to ensure safety and proper movement patterns. Additionally, visual feedback and oral feedback were consistently provided during each session to reinforce proprioceptive control and optimize neuromuscular adaptation.

### 2.5. Assessment of Outcomes

#### 2.5.1. Primary Outcomes

The CAIT score was the primary outcome and served as a validated measure of CAI of athletes. The CAIT is a nine-item self-reported questionnaire with a scale of 0 to 30 that is used to quantify CAI; lower scores indicate greater instability and higher scores reflect better stability [18]. The CAIT has demonstrated strong validity and reliability, thus effectively distinguishing individuals with and without CAI with high test–retest reliability (intraclass correlation coefficient = 0.93) and internal consistency (Cronbach’s α = 0.845–0.878) [18].

In addition to the CAIT score, the results of the T-test, lateral hop test, and figure-8 hop test were evaluated as primary outcomes. These tests were specifically selected on the basis of their relevance to basketball-specific performance demands. The *T*-test effectively assesses multidirectional agility, lateral quickness, and rapid directional changes, all of which are fundamental to basketball performance [19]. Similarly, the lateral hop test evaluates lateral movement stability and rapid weight-shifting ability, reflecting typical side-to-side movements encountered in basketball defense and offense [15]. The figure-8 hop test further measures dynamic single-leg stability, proprioceptive control, and the athlete’s capacity to maintain balance during complex movement patterns resembling typical basketball maneuvers [20]. Together, these tests provide a comprehensive assessment of functional ankle stability within sport-specific contexts relevant to basketball players with CAI.

The *T*-test was performed with four cones arranged in a T-shape, with a central cone 9.14 m from the starting point and two additional cones placed 4.57 m apart. Participants sprinted forward, shuffled laterally, and backpedaled; they touched each cone as required [19]. Times were recorded using a stopwatch. Each participant completed one practice trial and two measured trials, and the fastest time was used for the analysis. Trials were invalid if participants failed to touch the cones, crossed their legs while shuffling, or did not maintain forward-facing movement.

The lateral hop test required participants to stand on one leg behind a marked line and perform continuous lateral hops over a 30 cm distance for 10 s. Participants were instructed to hop as quickly and consistently as possible while maintaining balance. The total number of successful hops was recorded; incomplete jumps—such as those involving loss of balance, touching the ground with the opposite foot, or failing to clear the distance—were not included [11].

The figure-8 hop test involved hopping on one leg in a figure-8 pattern around two cones placed 5 m apart. Participants completed two trials, and the fastest lap time was recorded using a stopwatch. Trials were invalidated if participants lost balance, touched the ground with the opposite foot, or deviated from the designated course [11].

All primary outcomes were assessed in an indoor setting on a wooden gymnasium floor, and all participants wore athletic shoes. Assessments were conducted at baseline (before the intervention) and at 6 weeks after the intervention.

#### 2.5.2. Secondary Outcomes

Secondary outcomes were assessed to evaluate functional outcomes, including static balance, dynamic balance, and ankle strength. Static balance was measured during a 10 s single-leg stance with the eyes closed on a force plate (K-Force plate^®^; KINVENT, Montpellier, France). Balance stability was analyzed using the ellipse area and velocity of the center of pressure. Participants stood on the force plate with the hands folded across the chest and the opposite leg held at 30° hip flexion and 45° knee flexion; they maintained their stance with the eyes closed for 10 s. Trials were repeated if the opposite leg touched the ground or balance was lost [11].

Dynamic balance in the anterior, posterolateral, and posteromedial directions was assessed using the Star Excursion Balance Test. Participants completed four familiarization trials followed by three recorded attempts in each direction. Standing with the hallux of the tested limb at the Star Excursion Balance Test grid center and with the hands on the hips, participants extended the opposite leg as far as possible. Trials were invalid if participants lost balance, lifted a hand, moved the stance foot, or failed to return to the starting position. The highest reach distance in each direction was recorded. The composite Star Excursion Balance Test score was calculated by summing the maximum reach distances, normalizing the sum to the limb length, and expressing the sum as a percentage. Limb length was measured from the anterior superior iliac spine to the medial malleolus in the supine position [21].

Ankle strength was measured using a handheld dynamometer (K-Push^®^; KINVENT, Montpellier, France) with high intra-rater and inter-rater reliability to assess isometric plantar flexion, dorsiflexion, inversion, and eversion [22]. Participants were seated on an examination table with the legs fully extended and secured to prevent movement, thus ensuring that the ankle remained in a subtalar neutral position. The handheld dynamometer was applied at the following specific anatomical landmarks: the plantar surface from the first to fifth metatarsal heads (plantar flexion); the dorsal surface from the first to fifth metatarsal heads (dorsiflexion); the lateral aspect of the fifth metatarsal head (eversion); and the medial aspect of the first metatarsal head (inversion). Each strength measurement was repeated three times, with 10 s of rest allowed between trials to minimize fatigue.

### 2.6. Statistical Analysis

All statistical analyses were performed using SPSS software (version 23.0; IBM Corp., Armonk, NY, USA). The normality of data distributions was assessed using the Shapiro–Wilk test. For continuous variables that met the normality assumption, an independent *t* test was used to examine baseline homogeneity; however, height was analyzed using the Mann–Whitney U test because of its non-normal distribution. Categorical variables were assessed using Fisher’s exact test.

To evaluate differences between groups over time, a two-way repeated measures analysis of variance was conducted to assess interaction effects (group × time). Post hoc analyses for within-group changes were performed using a simple effect analysis.

Effect sizes for the repeated measures analysis were calculated using partial eta squared ($\eta_{p}^{2}$). The resulting values were interpreted as follows: small effect, 0.01 ≤ $\eta_{p}^{2}$ < 0.06; medium effect, 0.06 ≤ $\eta_{p}^{2}$ < 0.14; and large effect, $\eta_{p}^{2}$ ≥ 0.14. The level of significance was set at *p* < 0.05 for all analyses.

## 3. Results

In total, 32 adolescent female basketball players from eight teams were enrolled, and all participants completed the study without any dropouts. Teams were cluster-randomized, with four teams allocated to the hop-stabilization training group (HG; *n* = 16) and four teams to the balance training group (BG; *n* = 16).

In the HG, 17 players of 65 total team members met the criteria for CAI, of whom 1 was excluded based on the eligibility criteria. In the BG, 19 players of 58 team members were identified to have CAI, and 2 were excluded accordingly. There were no significant differences in baseline characteristics or pre-intervention outcome measures between the two groups (*p* > 0.05), indicating appropriate group homogeneity. The baseline characteristics of the participants are presented in Table 2.

### 3.1. Primary Outcomes

**Figure 2:**
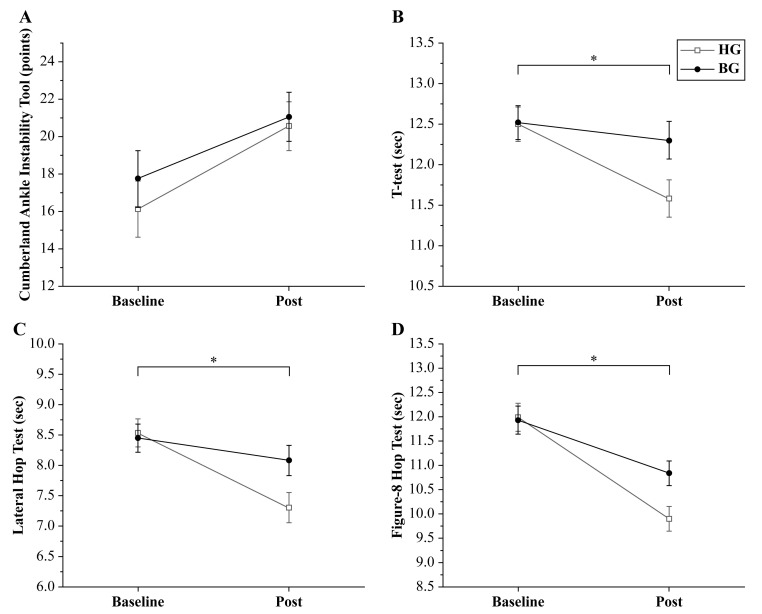
Changes in primary outcomes between baseline and after the intervention (Post) of the hop-stabilization training group (HG) and balance training group (BG). (**A**) Cumberland ankle instability tool (CAIT) scores improved in both groups; a significant group × time interaction was not observed. The (**B**) *t*-test, (**C**) lateral hop test, and (**D**) figure-8 hop test results revealed a significant time × group interaction effect. The improvements of the HG were greater than those of the BG (* *p* < 0.05). Error bars indicate the standard error of the mean (SEM).

The between-group results of the primary outcomes are illustrated in Figure 2. The CAIT did not exhibit a significant time × group interaction effect (*p* = 0.424; *Δ* = −0.50; 95% confidence [CI]: −4.28 to 3.28; $\eta_{p}^{2}$ = 0.021). However, a significant main effect of time (*p* < 0.001) that supported the post hoc analysis of within-group differences was observed. Significant improvements were subsequently observed in both the HG (*p* < 0.001) and BG (*p* = 0.002).

The *T*-test demonstrated a significant time × group interaction effect (*p* = 0.007; *Δ* = −0.72; 95% CI: −1.38 to −0.06; $\eta_{p}^{2}$ = 0.218). The within-group analysis showed a significant improvement in the HG after the intervention (*p* < 0.001), whereas the BG did not exhibit a statistically significant difference (*p* = 0.216).

The lateral hop test also revealed a significant time × group interaction effect (*p* = 0.027; *Δ* = −0.78; 95% CI: −1.51 to −0.05; $\eta_{p}^{2}$ = 0.153). The within-group analysis showed significant improvement in the HG after the intervention (*p* < 0.001), whereas the BG did not demonstrate a statistically significant difference (*p* = 0.170).

Similarly, the figure-8 test demonstrated a significant time × group interaction effect (*p* = 0.002; *Δ* = −0.94; 95% CI: −1.66 to −0.22; $\eta_{p}^{2}$ = 0.283). The within-group analysis showed significant improvements in both the HG and BG after the intervention (*p* < 0.001 for both groups). A comprehensive summary of the primary outcomes is presented in Table 3.

### 3.2. Secondary Outcomes

**Figure 3:**
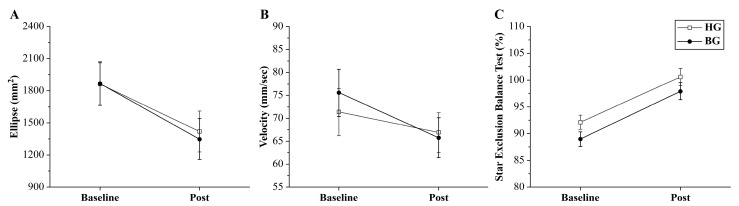
Changes in static and dynamic balance measures. Both groups experienced improvements in the center of pressure (COP) ellipse area (**A**), COP velocity (**B**), and Star Excursion Balance Test (SEBT) results (**C**). A significant time × group interaction effect was not observed. Error bars indicate the standard error of the mean (SEM). HG: hop-stabilization training group; BG: balance training group; Post: after the intervention.

**Figure 4:**
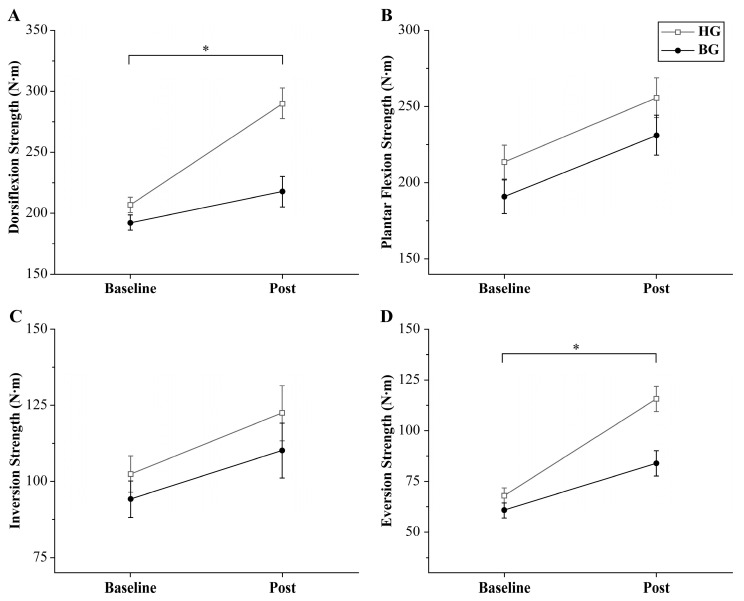
Changes in isometric ankle strength. Significant time × group interaction effects were observed for dorsiflexion (**A**) and eversion strength (**D**) (* *p* < 0.05). Significant time × group interaction effects for plantar flexion (**B**) and inversion strength (**C**) were not observed. Error bars indicate the standard error of the mean (SEM). HG: hop-stabilization training group; BG: balance training group; Post: after the intervention.

The between-group balance outcomes are illustrated in Figure 3, and the strength outcomes are presented in Figure 4. For static balance, the ellipse area did not show a significant time × group interaction effect (*p* = 0.771; *Δ* = −71.03; 95% CI: −578.97 to 592.17; $\eta_{p}^{2}$ = 0.003). However, a significant main effect of time (*p* = 0.021) that supported the post hoc analysis of within-group differences was observed. Significant improvements were subsequently observed in both the HG (*p* = 0.024) and BG (*p* = 0.009). Similarly, velocity did not exhibit a significant time × group interaction effect (*p* = 0.412; *Δ* = 1.14; 95% CI: −11.37 to 13.65; $\eta_{p}^{2}$ = 0.023). However, a significant main effect of time (*p* = 0.034) that supported the post hoc analysis of within-group differences was observed. The within-group analysis revealed no statistically significant difference in the HG after the intervention (*p* = 0.335), whereas the BG showed a significant improvement (*p* = 0.039).

Regarding the Star Excursion Balance Test, a significant time × group interaction effect was not observed (*p* = 0.818; *Δ* = 2.67; 95% CI: −1.94 to 7.28; $\eta_{p}^{2}$ = 0.002). However, a significant main effect of time (*p* < 0.001) that supported the post hoc analysis of within-group differences was observed. The within-group analysis showed that both the HG and BG experienced significant improvement after the intervention (*p* < 0.001 for both groups).

Regarding ankle strength, dorsiflexion demonstrated a significant time × group interaction effect (*p* = 0.001; *Δ* = 72.24; 95% CI: 35.60–108.89; $\eta_{p}^{2}$ = 0.294). The within-group analysis showed significant improvements in both the HG and BG after the intervention (*p* < 0.001 and *p* = 0.035, respectively). In contrast, plantar flexion did not exhibit a significant interaction effect (*p* = 0.915; *Δ* = 24.73; 95% CI: −13.00 to 62.46; $\eta_{p}^{2}$ < 0.001). However, a significant main effect of time (*p* < 0.001) that supported the post hoc analysis of within-group differences was observed. Both groups showed significant improvements after the intervention (*p* = 0.005 and *p* = 0.007, respectively).

Regarding inversion strength, a significant time × group interaction effect was not observed (*p* = 0.730; *Δ* = −12.26; 95% CI: −38.35 to 13.84; $\eta_{p}^{2}$ = 0.063). However, a significant main effect of time (*p* < 0.001) that supported the post hoc analysis of within-group differences was observed. A subsequent analysis revealed a statistically significant improvement in the HG (*p* = 0.005), whereas the BG did not exhibit a significant change (*p* = 0.064). Conversely, eversion strength demonstrated a significant time × group interaction effect (*p* = 0.010; *Δ* = 31.69; 95% CI: 13.50–49.88; $\eta_{p}^{2}$ = 0.200). The within-group analysis indicated significant improvements in both the HG and BG after the intervention (*p* < 0.001 for both groups). A detailed summary of the secondary outcomes is presented in Table 4.

## 4. Discussion

This study aimed to compare the effects of hop-stabilization training (hop training) and those of traditional balance training on adolescent female basketball players with CAI. Our results revealed that although both interventions improved self-reported ankle stability and balance measures, the HG exhibited significantly greater enhancements in the results of dynamic performance tests (the T-test, lateral hop test, and figure-8 hop test) as well as notable improvements in ankle strength, particularly in dorsiflexion and eversion, compared to those of the BG. These findings hold significant practical implications for sports practice, particularly in basketball, where repetitive jumping, landing, and rapid directional changes are frequently required. Improved functional ankle stability through hop training directly translates into a reduced risk of recurrent ankle injuries by enhancing shock absorption capability and dynamic balance during high-impact movements. Consequently, athletes may experience fewer interruptions from injuries, allowing them to sustain consistent training intensity and competitive performance. Therefore, coaches and sports professionals are encouraged to integrate hop exercises into regular training regimens, because this approach not only facilitates neuromuscular adaptations crucial for injury prevention but also substantially enhances overall agility and movement efficiency on the court.

Moreover, these findings support our hypothesis and highlight that sport-specific training, such as hop training, effectively enhances functional performance by closely replicating the dynamic and multidirectional demands encountered in basketball. Consequently, this targeted training approach may facilitate practical improvements in on-court agility, stability, and performance efficiency, thereby directly benefiting athletes and providing valuable insights for coaches and sports rehabilitation professionals.

Ankle instability not only leads to abnormal biomechanics during basketball movements but also results in actual performance losses during competitive play. Wang et al. [23] highlighted how CAI alters joint biomechanics during key basketball maneuvers, such as the three-step layup, leading to compromised movement efficiency and increased injury risk. Similarly, Ceasar et al. [24] demonstrated that both knee and ankle injuries negatively impacted the performance of National Basketball Association players after injury.

Based on these findings, managing ankle instability of basketball players is essential to preventing injuries and minimizing performance losses. Additionally, our findings indicate that both hop training and balance training effectively improved ankle stability of adolescent female basketball players with CAI.

These improvements in CAI may be attributable to enhancements in both static and dynamic balance capacities. In the present study, these balance parameters were evaluated as secondary outcomes, with both demonstrating significant improvements after the intervention. Enhanced static balance, as measured by the reduced ellipse area on the force plate, may reflect improved proprioceptive function and postural control during stationary tasks [25]. However, gains in dynamic balance may facilitate more effective neuromuscular control during sport-specific, multidirectional movements [26]. These findings are supported by those of previous research. Laddawong et al. [16] reported that hop training significantly improved dynamic balance and ankle stability of collegiate athletes with CAI and demonstrated that a hop-stabilization warm-up program enhanced neuromuscular control, ground reaction forces, and muscle activity during cutting movements, which are essential to athletic performance. Similarly, Minoonejad et al. [15] conducted a randomized controlled trial and found that hop training effectively improved the ankle stability of basketball players with CAI. Furthermore, Park et al. [11] reported that balance training significantly enhanced both the postural control and ankle stability of elite adolescent male soccer players with ankle instability. These findings underscore the efficacy of balance training as a targeted intervention that can improve sensorimotor deficits and facilitate rehabilitation.

In the present study, the HG demonstrated superior performance compared to that of the BG during the T-test, lateral hop, and figure-8 tests. These findings can be primarily attributed to the principle of specificity inherent in the hop training intervention, which closely simulates the multidirectional and dynamic demands encountered during basketball. Hop training emphasizes controlled landing mechanics and rapid directional changes, thereby enhancing neuromuscular control and optimizing biomechanical alignment during high-impact movements [14]. Such improvements in landing mechanics are critical to executing complex athletic tasks because they facilitate more effective force absorption and redistribution, ultimately contributing to enhanced dynamic balance and increased ankle strength, particularly in dorsiflexion and eversion. Previous investigations have corroborated these findings. Ardakani et al. [14] demonstrated that hop training significantly improved landing biomechanics and neuromuscular control of individuals with CAI. Minoonejad et al. [15] reported that similar training protocols led to marked improvements in dynamic performance metrics among basketball players with CAI. Moreover, Laddawong et al. [16] indicated that a hop-stabilization warm-up program acutely enhanced dynamic balance, ground reaction force, and muscle activity during cutting movements of collegiate athletes with CAI. Collectively, these studies support the notion that the sport-specific and neuromuscular adaptations elicited by hop training are more effective than those provided by traditional balance training for mitigating the functional deficits associated with CAI, thereby offering a plausible explanation of the superior performance outcomes observed during agility and dynamic hopping assessments.

Both balance and hop training protocols can enhance overall ankle stability as well as static and dynamic balance. However, the extant literature suggested that traditional balance training may have a limited capacity to improve dynamic functional outcomes, particularly those involving rapid multidirectional movements. For example, Liu et al. [20] conducted a meta-analysis of unstable surface training for CAI and found that although balance training led to significant improvements in static performance measures (such as time-in-balance and foot-lift test results) and specific directions of the Star Excursion Balance Test, it did not result in marked enhancement of the hop function, as evaluated by side-hop and figure-8 hop tests. Therefore, although balance training effectively promotes proprioceptive gains and postural control, it may not provide a sufficient stimulus to induce the neuromuscular adaptations necessary for executing explosive sport-specific movements, such as those commonly encountered in basketball.

Considering the limited impact of traditional balance training on hop performance and dynamic agility, hop training is a more comprehensive rehabilitation strategy for adolescent female basketball players with CAI. This modality not only augments both static and dynamic balance parameters but also produces significant improvements in functional performance, thereby potentially mitigating the risk of injury during high-impact sport-specific activities.

In summary, the present study demonstrated that hop training yielded significantly greater improvements in dynamic performance measures as well as notable enhancements in ankle strength compared to those achieved with traditional balance training for adolescent female basketball players with CAI. These findings support the principle of specificity, indicating that training interventions that more closely mimic the rapid, multidirectional, and high-impact demands of basketball more effectively promote functional improvements and neuromuscular adaptations compared to those provided by conventional balance exercises that predominantly target static and basic dynamic stability.

Despite these promising results, this study had several limitations. First, the sample size was relatively small; therefore, the generalizability of our findings is restricted. Additionally, the 6-week intervention period precluded conclusions regarding the long-term sustainability of the observed improvements. Second, the cluster randomization approach, although useful for minimizing cross-contamination between participants, may have introduced variability because of inherent differences among basketball teams that were not fully controlled. Third, the reliance on self-reported measures (e.g., CAIT score) may not have fully captured the complex in-game functional demands experienced by athletes. Moreover, external factors such as differences in training history and biomechanical variability were not exhaustively controlled.

Future research should aim to address these limitations by incorporating larger sample sizes, extending the duration of interventions and follow-up assessments, and using a more comprehensive array of outcome measures, including ecological validity assessments in competitive environments, to elucidate the mechanisms underlying the superior benefits of hop training. Overall, the findings suggest that hop training is a promising rehabilitation and performance enhancement strategy for adolescent female basketball players with CAI that merits further investigations comprising more rigorously designed trials.

## 5. Conclusions

This study investigated the comparative effects of hop-stabilization training (hop training) and traditional balance training on adolescent female basketball players with CAI. Both intervention protocols produced significant improvements in ankle stability, as evidenced by enhanced CAIT scores and favorable changes in static and dynamic balance measures. However, hop training yielded superior improvements in sport-specific performance parameters. Specifically, the HG exhibited significantly greater enhancements in agility and dynamic hopping performance, as reflected by the results of the T-test, lateral hop test, and figure-8 hop test, compared to those exhibited by the BG. Moreover, improvements in ankle strength, particularly in dorsiflexion and eversion, were more pronounced in the HG, suggesting that, compared to traditional balance training, hop training may more effectively target the neuromuscular and proprioceptive deficits associated with CAI.

The findings of the present study support the incorporation of hop training into rehabilitation programs for adolescent female basketball players with CAI. By more closely replicating the dynamic and multidirectional demands of basketball, hop training appears to enhance functional performance beyond what is achievable with conventional balance training alone. Future research involving larger sample sizes and longer follow-up periods should aim to confirm these results and further explore the mechanisms underlying the superior benefits of hop training for this population.

## Figures and Tables

**Table 1:** Hop-stabilization training program.

Week	Exercises	Volume	Focus of Landing Mechanics	Tempo
Week 1	Side-to-side hops, forward and backward hops, figure-8 hops, zigzag hops, four-square hops	160	Knee alignment, flexed knees, shoulder-width stance	Not specified
Week 2	Same as week 1	200	Stability upon landing before the next hop	Not specified
Week 3	Same as week 1	240	Stability, alignment, controlled knee flexion	Not specified
Week 4	Same as week 1	320	Stability, alignment, knee control	2 Hz (metronome-guided, 2 hops/s)
Week 5	Same as week 1	320	Stability, alignment, knee control	2 Hz (metronome-guided, 2 hops/s)
Week 6	Same as week 1	300 (slight reduction for fatigue management)	Stability, alignment, controlled knee flexion	2 Hz (metronome-guided, 2 hops/s)

**Table 2:** Baseline characteristics (*n* = 32) ^1^.

	HG (*n* = 16)	BG (*n* = 16)	*p* Value
Age (years)	17.06 (0.85)	16.81 (0.75)	0.389 ^1^
Height (cm)	171.75 (7.33)	169.06 (6.90)	0.294 ^2^
Weight (kg)	66.25 (7.33)	64.25 (7.32)	0.323 ^1^
BMI (kg/m^2^)	22.5 (2.58)	22.46 (1.76)	0.963 ^1^
Right ankle (n)	7 (43.75%)	9 (56.25%)	0.724 ^3^
Left ankle (n)	9 (46.25%)	7 (43.75%)	
CAIT (points)	16.13 (6.83)	17.75 (5.17)	0.454 ^1^
*T*-test (s)	12.5 (0.59)	12.52 (1.01)	0.954 ^1^
Lateral hop (s)	8.53 (1.04)	8.45 (0.79)	0.802 ^1^
Figure-8 hop (s)	11.99 (0.92)	11.93 (1.33)	0.878 ^1^
Ellipse (mm^2^)	1864.61 (662.76)	1871.21 (936.02)	0.982 ^1^
Velocity (mm/s)	71.41 (17.68)	75.62 (23.16)	0.567 ^1^
SEBT (%)	92.14 (4.53)	88.98 (6.29)	0.114 ^1^
DF strength (N·m)	206.67 (29.08)	192.17 (19.83)	0.110 ^1^
PF strength (N·m)	213.42 (39.92)	190.81 (49.14)	0.164 ^1^
IV strength (N·m)	102.39 (26.25)	94.23(21.17)	0.341 ^1^
EV strength (N·m)	68 (15.42)	60.83 (14.55)	0.186 ^1^

HG: hop-stabilization training group; BG: balance training group; BMI: body mass index; CAIT: Cumberland ankle instability tool; CI: confidence interval; DF: dorsiflexion; EV: eversion; HG: hop-stabilization training group; IV: inversion; PF: plantar flexion; SEBT: Star Excursion Balance Test. Data are expressed as the mean (standard deviation). ^1^ Independent *t* test. ^2^ Mann–Whitney *U* test. ^3^ Fisher’s exact test.

**Table 3:** Primary outcomes.

	HG (*n* = 16)			BG (*n* = 16)		
	Baseline	After the Intervention	Mean Change (95% CI) ^1^	Baseline	After the Intervention	Mean Change (95% CI) ^1^
CAIT (points)	16.13 (1.51)	20.56 (1.31)	4.44 *** (2.44–6.44)	17.75 (1.51)	21.06 (1.31)	3.31 ** (1.31–5.31)
*t*-test (s)	12.5 (0.21)	11.58 (0.23)	−0.92 *** (−1.27 to −0.57)	12.52 (0.21)	12.3 (0.23)	−0.22 ** (−0.57 to 0.13)
Lateral hop (s)	8.53 (0.23)	7.3 (0.25)	−1.23 *** (−1.76 to −0.69)	8.45 (0.23)	8.08 (0.25)	−0.37 (−0.90 to 0.17)
Figure-8 hop (s)	11.99 (0.29)	9.90 (0.25)	−2.09 *** (−2.51 to −1.67)	11.93 (0.29)	10.84 (0.25)	−1.08 *** (−1.50 to −0.66)

HG: hop-stabilization training group; BG: balance training group; CAIT: Cumberland ankle instability tool; CI: confidence interval; HG: hop-stabilization training group. Data are presented as the estimated marginal mean (standard error). ^1^ Simple main effect analysis: ** *p* < 0.01, and *** *p* < 0.001.

**Table 4:** Secondary outcomes.

	HG (*n* = 16)			BG (*n* = 16)		
	Baseline	After the Intervention	Mean Change (95% CI) ^1^	Baseline	After the Intervention	Mean Change (95% CI) ^1^
Ellipse (mm^2^)	1864.61 (202.75)	1420.15 (192.04)	−444.46 * (−825.37 to −63.56)	1871.21 (202.75)	1349.12 (192.04)	−522.09 ** (−903.00 to −141.19)
Velocity (mm/s)	71.41 (5.15)	66.94 (4.33)	−4.46 (−13.76 to 4.83)	75.62 (5.15)	65.80 (4.33)	−9.82 * (−19.11 to −0.53)
SEBT (%)	92.14 (1.37)	100.61 (1.6)	8.48 *** (−11.49 to −5.46)	88.98 (1.37)	97.94 (1.60)	8.96 *** (−11.97 to −5.95)
DF strength (N·m)	206.67 (6.22)	289.96 (12.69)	83.29 *** (59.72–106.87)	192.17 (6.22)	217.72 (12.69)	25.55 * (1.98–49.12)
PF strength (N·m)	213.42 (11.19)	255.76 (13.06)	42.34 ** (13.73–70.95)	190.81 (11.19)	231.03 (13.06)	40.21 ** (11.60–68.82)
IV strength (N·m)	102.39 (5.96)	122.44 (9.03)	20.06 * (3.09–37.02)	94.23 (5.96)	110.19 (9.03)	15.96 (−1.01 to 32.92)
EV strength (N·m)	68.00 (3.75)	115.64 (6.30)	47.64 *** (34.70–60.59)	60.83 (3.75)	83.95 (6.30)	23.12 ** (10.18–36.06)

BG: balance training group; CI: confidence interval; DF: dorsiflexion; EV: eversion; HG: hop-stabilization training group; IV: inversion; PF: plantar flexion; SEBT: Star Excursion Balance Test. Data are expressed as the estimated marginal mean (standard error). ^1^ Simple main effect analysis: * *p* < 0.05, ** *p* < 0.01, and *** *p* < 0.001.

## Data Availability

The data presented in this study are openly available in Zenodo at https://doi.org/10.5281/zenodo.15120700 (accessed on 1 April 2025).

## Abbreviations

The following abbreviations are used in this manuscript:

BG	Balance training group
CAI	Chronic ankle instability
CAIT	Cumberland ankle instability tool
HG	Hop-stabilization training group

## References

[1] Pasanen K., Ekola T., Vasankari T., Kannus P., Heinonen A., Kujala U.M., Parkkari J. (2017). High ankle injury rate in adolescent basketball: A 3-year prospective follow-up study. Scand. J. Med. Sci. Sports.

[2] Herzog M.M., Kerr Z.Y., Marshall S.W., Wikstrom E.A. (2019). Epidemiology of ankle sprains and chronic ankle instability. J. Athl. Train..

[3] Biz C., Nicoletti P., Tomasin M., Bragazzi N.L., Di Rubbo G., Ruggieri P. (2022). Is Kinesio taping effective for sport performance and ankle function of athletes with chronic ankle instability (CAI)? A systematic review and meta-analysis. Medicina.

[4] Lin C.-I., Houtenbos S., Lu Y.-H., Mayer F., Wippert P.-M. (2021). The epidemiology of chronic ankle instability with perceived ankle instability—A systematic review. J. Foot Ankle Res..

[5] Sarcon A.K., Heyrani N., Giza E., Kreulen C. (2019). Lateral ankle sprain and chronic ankle instability. Foot Ankle Orthop..

[6] Liu X., He M., Hu R., Chen Z. (2024). Randomized controlled trial study of intelligent rehabilitation training system for functional ankle instability. Sci. Rep..

[7] Chan L.Y.T., Sim Y.T.N., Gan F.K., Bin Abd Razak H.R.B. (2022). Effect of chronic ankle instability on lower extremity kinematics, dynamic postural stability, and muscle activity during unilateral jump-landing tasks: A systematic review and meta-analysis. Phys. Ther. Sport.

[8] Yılmaz O., Soylu Y., Erkmen N., Kaplan T., Batalik L. (2024). Effects of proprioceptive training on sports performance: A systematic review. BMC Sports Sci. Med. Rehabil..

[9] Hiller C.E., Kilbreath S.L., Refshauge K.M. (2011). Chronic ankle instability: Evolution of the model. J. Athl. Train..

[10] Mollà-Casanova S., Inglés M., Serra-Añó P. (2021). Effects of balance training on functionality, ankle instability, and dynamic balance outcomes in people with chronic ankle instability: Systematic review and meta-analysis. Clin. Rehabil..

[11] Park H.S., Oh J.K., Kim J.Y., Yoon J.H. (2024). The effect of strength and balance training on kinesiophobia, ankle instability, function, and performance in elite adolescent soccer players with functional ankle instability: A prospective cluster randomized controlled trial. J. Sports Sci. Med..

[12] Hall E.A., Chomistek A.K., Kingma J.J., Docherty C.L. (2018). Balance- and strength-training protocols to improve chronic ankle instability deficits, Part I: Assessing clinical outcome measures. J. Athl. Train..

[13] Su Y., Li W., Pan C., Shi Y. (2024). Effects of combination of strength and balance training on postural control and functionality in people with chronic ankle instability: A systematic review and meta analysis. BMC Sports Sci. Med. Rehabil..

[14] Ardakani M.K., Wikstrom E.A., Minoonejad H., Rajabi R., Sharifnezhad A. (2019). Hop-stabilization training and landing biomechanics in athletes with chronic ankle instability: A randomized controlled trial. J. Athl. Train..

[15] Minoonejad H., Karimizadeh Ardakani M.K., Rajabi R., Wikstrom E.A., Sharifnezhad A. (2019). Hop stabilization training improves neuromuscular control in college basketball players with chronic ankle instability: A randomized controlled trial. J. Sport Rehabil..

[16] Laddawong T., Saito H., Soga T., Hirose N. (2024). Acute effects of a hop-stabilization warm-up program on dynamic balance, ground reaction force, and muscle activity during cutting movements in collegiate athletes with chronic ankle instability. Int. J. Exerc. Sci..

[17] Wright C.J., Arnold B.L., Ross S.E., Linens S.W. (2014). Recalibration and validation of the Cumberland ankle instability tool cutoff score for individuals with chronic ankle instability. Arch. Phys. Med. Rehabil..

[18] Wang W., Liao D., Kang X., Zheng W., Xu W., Chen S., Xie Q. (2021). Development of a valid Chinese version of the Cumberland Ankle Instability Tool in Chinese-speaking patients with chronic ankle instability disorders. Sci. Rep..

[19] Munro A.G., Herrington L.C. (2011). Between-session reliability of four hop tests and the agility T-test. J. Strength Cond. Res..

[20] Liu S., Gou B., Zhao Z., Wang Q. (2024). Exploratory analysis of unstable surface training: A systematic review and meta-analysis for chronic ankle instability. Arch. Rehabil. Res. Clin. Transl..

[21] Picot B., Terrier R., Forestier N., Fourchet F., McKeon P.O. (2021). The star excursion balance test: An update review and practical guidelines. Int. J. Athl. Ther. Train..

[22] Olds M., McLaine S., Magni N. (2023). Validity and reliability of the Kinvent handheld dynamometer in the athletic shoulder test. J. Sport Rehabil..

[23] Wang L., Ye J., Zhang X. (2023). Ankle biomechanics of the three-step layup in a basketball player with chronic ankle instability. Sci. Rep..

[24] Ceasar J., Yeich A., Shafeek P., Kumar K., Olympia R.P. (2024). The impact of knee and ankle injuries on National Basketball Association player performance post-injury. Cureus.

[25] Chen B., Liu P., Xiao F., Liu Z., Wang Y. (2021). Review of the upright balance assessment based on the force plate. Int. J. Environ. Res. Public Health.

[26] Zech A., Hübscher M., Vogt L., Banzer W., Hänsel F., Pfeifer K. (2010). Balance training for neuromuscular control and performance enhancement: A systematic review. J. Athl. Train..

